# Striving toward safe abortion services in Nepal: A review of barriers and facilitators

**DOI:** 10.1002/hsr2.1877

**Published:** 2024-02-22

**Authors:** Alok Atreya, Kishor Adhikari, Samata Nepal, Milan Bhusal, Ritesh G. Menezes, Dhan B. Shrestha, Deepak Shrestha

**Affiliations:** ^1^ Department of Forensic Medicine Lumbini Medical College Palpa Nepal; ^2^ Department of Community Medicine, School of Public Health Chitwan Medical College Bharatpur Nepal; ^3^ Department of Community Medicine Lumbini Medical College Palpa Nepal; ^4^ Medical Officer Gulmi Hospital Tamghas, Gulmi Nepal; ^5^ Department of Pathology, College of Medicine Imam Abdulrahman Bin Faisal University Dammam Saudi Arabia; ^6^ Department of Internal Medicine Mount Sinai Hospital Chicago Illinois USA; ^7^ Department of Obstetrics and Gynaecology Lumbini Medical College Palpa Nepal

**Keywords:** family planning services, health facilities, pregnancy, rural population

## Abstract

**Background and Aims:**

Despite the decriminalization of abortion in Nepal in 2002, unsafe abortion is still a significant contributor to maternal morbidity and mortality. Nepal has witnessed a significant drop in abortion‐related severe complications and maternal deaths owing to the legalization of abortion laws, lowered financial costs, and wider accessibility of safe abortion services (SAS). However, various factors such as sociocultural beliefs, financial constraints, geographical difficulties, and stigma act as barriers to the liberal accessibility of SAS. This review aimed to determine key barriers obstructing women's access to lawful, safe abortion care and identify facilitators that have improved access to and quality of abortion services.

**Methods:**

A systematic search strategy utilizing the databases PubMed, CINAHL, Scopus, and Embase was used to include studies on the accessibility and safety of abortion services in Nepal. Data were extracted from included studies through close reading. Barriers and facilitators were then categorized into various themes and analyzed.

**Results:**

Of 223 studies, 112 were duplicates, 73 did not meet the inclusion criteria, and 18 did not align with the research question; thus, 20 studies were included in the review. Various barriers to SAS in Nepal were categorized as economic, geographic, societal, legal/policy, socio‐cultural, health systems, and other factors. Facilitators improving access were categorized as economic/geographic/societal, legal/policy, socio‐cultural, and health systems factors. The patterns and trends of barriers and facilitators were analyzed, grouping them under legal/policy, socio‐cultural, geographic/accessibility, and health systems factors.

**Conclusion:**

The review identifies financial constraints, unfavorable geography, lack of infrastructure, and social stigmatization as major barriers to SAS. Economics and geography, legalization, improved access, reduced cost and active involvement of auxiliary nurse‐midwives and community health volunteers are key facilitators.

## Key points

- The legalization of abortion in Nepal has not ensured universal access to safe services.
- Stigma, lack of awareness, and negative attitude of providers hamper access.
- Rural, low‐income women face additional obstacles such as distance, cost and lack of confidentiality.

## 1 INTRODUCTION

Terminating a pregnancy before full term is reached is known as abortion. When the reasons for having an abortion do not align with legal justifications, it often results in women resorting to secret and unsafe abortions. In Nepal, unsafe abortion is considered a significant contributor to maternal morbidity and mortality. This can lead to serious complications such as injuries, hemorrhage, and sepsis, that can have a long‐term detrimental effect on the mother's health. Additionally, using risky abortion methods may increase the chance of developing secondary infertility.^1^, ^2^, ^3^, ^4^ Abortion was illegal in Nepal until 2002. In Nepal, approximately half of all maternal deaths caused by complications from unsafe abortions.^5^, ^6^, ^7^, ^8^, ^9^ Despite strict laws, a large number of unsafe abortions occur each year, reflected by the fact that one‐third of all imprisoned women and half of all hospitalized women were due to the consequences of illegal termination of pregnancies.^10^, ^11^ Following amendments to the Penal Code in 2002, abortion was permitted on certain grounds.^12^, ^13^, ^14^ Under current law, a woman has the right to an abortion for any reason within the first 12 weeks of pregnancy, and up to 18 weeks in cases of sexual assault.^15^ Additionally, abortion at any gestational age is legally permitted if a doctor certifies that continued pregnancy endangers pregnant woman's physical and/or mental health; or that the fetus is deformed.^5^, ^15^ In 2018, a new law known as the Safe Motherhood and Reproductive Health Rights Act (SMRHR) was enacted by Parliament to increase women's access to safe and legal abortion.^16^

In Nepal, safe abortion services (SAS) are available at the federal, provincial, and municipal levels, as well as through outreach programs.^17^ Public sector health facilities in the country offer free abortion care to the client and receive reimbursement ranging from 800 to 3000 Nepalese rupees from their respective provincial governments based on the service provided.^17^ These multiple efforts to encourage SAS across have resulted in a significant decline in maternal mortality ratio, that is, from 539 maternal deaths per 100,000 live births in 1996^18^ to 151 per 100,000 live births in 2022.^19^ During the period from 2007 to 2010, a notable trend emerged: a significant reduction in the severity of complications caused by unsafe abortions.^7^

A study conducted in Nepal in 2014 analyzed the distribution of legal abortions across sectors and found that, approximately 37% occurred in public facilities, 34% in nongovernmental facilities, and the remaining 29% in private clinics.^9^ Interestingly, these statistics are influenced by the fact that unsafe abortion is the third most common cause of maternal mortality in Nepal.^9^

According to the data from the Ministry of Health and Population, in the fiscal year 2020/21, 79,952 women in Nepal utilized SAS. This represents a slight decrease from 87,869 women who used SAS in the previous fiscal year 2019/20; overall, SAS has shown a downward trend since a peak of 98,640 women accessing services in 2017/18.^17^

The global gag rule (GGR) prohibits non‐US‐based nongovernmental organizations (NGOs) from providing, referring, or counseling abortion services that receive US global health assistance. It also prohibits advocating for abortion law reforms.^20^ GGR has fragmented sexual and reproductive health (SRH) service delivery in many countries, including Nepal.^20^ The broad scope of this policy affects funding allocation, posing challenges even for NGOs without direct US government funding. This disruption is consistent with pre‐existing barriers to legal abortion, such as limited knowledge, stigma, cost, and limited access to services. In addition to abortion, GGR contributes to reducing contraceptive use, increases induced abortion, and has a chilling effect on advocacy efforts.^21^

This review aimed to identify barriers and facilitators affecting access to SAS in Nepal, focusing on major obstacles hindering women's access and identifying improvements in quality and accessibility.

## 2 METHODS

### 2.1 Research design

We utilized a systematic search strategy to identify relevant studies investigating the accessibility and safety of abortion services in Nepal.

### 2.2 Research question

The research question guiding the review was: “*What are the barriers and facilitators for accessing safe abortion services in Nepal*?”

#### 2.2.1 Inclusion criteria

The studies included in this review satisfied the following requirements: (i) they were conducted in Nepal, (ii) they offered insights into abortion accessibility and safety, and (iii) they were published in peer‐reviewed journals or recognized sources in the English language.

#### 2.2.2 Exclusion criteria

To ensure the accuracy of our analysis, we specifically chose to include only studies written in English that reported original research findings and published before March 31, 2023. Additionally, we excluded studies such as reviews, commentaries, editorials, and letters that did not meet our inclusion criteria.

### 2.3 Database searched

To systematically search for literature on abortion access and saftey in Nepal, a syntax was created combining the following keywords: abortion, access, safety, Nepal.

(("abortion" OR "terminat*" OR "miscarriage*") AND ("access*" OR "availability" OR "utilization") AND ("safety" OR "complication*" OR "risk*") AND ("Nepal" OR "Nepali"))

We then systematically searched four databases: PubMed, Embase, CINAHL, and Scopus. In addition to these databases, a search of the grey literature was also conducted, which included difficult‐to‐find sources such as conference proceedings, government reports, and dissertations. This search was carried out using the World Health Organization Global Health Library as well as other sources such as Nepalese medical journals and databases including the *Nepal Journal of Obstetrics and Gynecology* and *Nepal Journals Online* (*NepJoL*).

### 2.4 Data extraction

Identified studies underwent title/abstract and full‐text screening for eligibility. Data were extracted from included studies through close reading. Barriers and facilitators were categorized into themes. Patterns and trends were analyzed across themes to synthesize insights on factors impacting abortion safety and accessibility in Nepal.

### 2.5 Ethical clearance

Ethical clearence is not applicaple as this study reviewed published studies.

## 3 RESULTS

**Figure 1:**
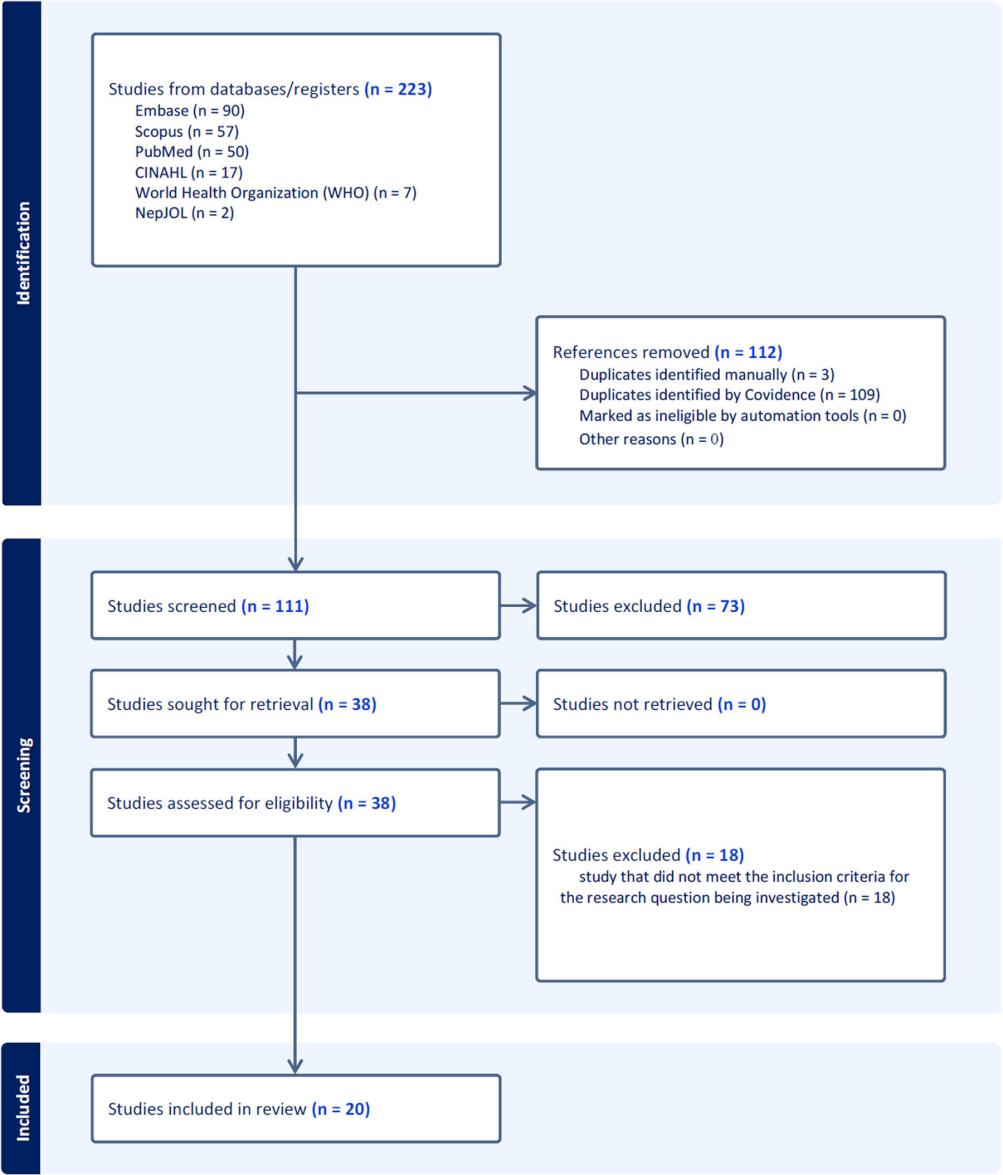
Flowchart of the review procedure.

A total of 223 studies were retrieved from the search of databases, of which 112 were removed as duplicates. The remaining 111 studies were then evaluated for the inclusion criteria, which led to the further exclusion of 73 studies. Subsequently, the full texts of 38 studies underwent evaluation for eligibility, ultimately resulting in the inclusion of 20 studies (Supporting Information S1: File 1) that aligned with the research question and met the specified inclusion criteria.^7^, ^8^, ^9^, ^22^, ^23^, ^24^, ^25^, ^26^, ^27^, ^28^, ^29^, ^30^, ^31^, ^32^, ^33^, ^34^, ^35^, ^36^, ^37^, ^38^ Flow diagram of the study design process is depicted in Figure 1.

We identified various barriers to SAS in Nepal, categorized as: economic, geographic, societal; legal/policy; sociocultural; healthcare systems; and other factors (Table 1). In the category of “economic, geographic, and social barriers,” major factors that barred access to SAS included financial constraints, high cost of abortion services in private clinics, limited availability of resources and infrastructure in rural areas, distance and transportation barriers, and lack of availability and accessibility to abortion services. The legal and policy barriers included a lack of knowledge about the law and SAS, fear of legal consequences, and legal restrictions. Other barriers include societal norms that stigmatize and discriminate against this form of healthcare, negative attitudes from healthcare providers, and cultural beliefs that perpetuate gender‐based power imbalances. Furthermore, inadequate knowledge and training of providers, limited privacy and confidentiality, and insufficient resources also contribute to hindering access to medical abortion.

**Table 1:** Barriers and their categories.

Barriers
Economic, geographic, and societal factors
Scarcity of resources and infrastructure in rural areas^8^, ^9^, ^25^, ^32^
Distance and transportation hindering access to service providers^30^, ^33^
Financial constraints^8^, ^34^
Limited availability and accessibility of service in rural areas^7^, ^24^
High cost of abortion services in private clinics^7^, ^23^, ^24^, ^25^, ^30^, ^33^
Legal and policy factors
Lack of knowledge of the law and availability of SAS^8^
Fear of legal consequences or social stigma for seeking an abortion^7^, ^8^
Legal restrictions^24^, ^34^, ^37^
Restricted legal status of abortion where access to safe care is limited^35^
A policy that bars women living more than 2 h from a health facility from using medical abortion^28^
Challenges in obtaining necessary documents and approvals^38^
Social and cultural factors
Stigma and discrimination against women seeking abortion^8^, ^25^, ^30^
Stigma surrounding abortion^32^, ^33^, ^38^
Negative attitudes from providers^28^, ^29^
Sociocultural factors such as cultural beliefs^34^
Sociocultural factors such as gender‐based power dynamics and lack of decision‐making power for women^34^
Fear of judgment or discrimination from healthcare providers or others in the community^23^, ^24^
Healthcare system factors
Lack of knowledge about abortion and available services^8^, ^22^, ^33^
Lack of trained providers^8^, ^34^
Inadequate knowledge and experience about the services required^23^
Lack of confidentiality and privacy in healthcare settings^7^, ^23^, ^35^
Inadequate capacity to meet the needs of the population^26^
Inadequate training and resources for healthcare providers^22^, ^25^
Limited availability of trained providers and facilities^8^, ^30^, ^38^
Lack of service providers and physician oversight^31^
Challenges in accessing effective postabortion contraception^34^
Inadequate or inaccurate information and unsafe medications provided by untrained pharmacy workers, and the lack of a formal referral mechanism^26^, ^35^
Other factors
Lack of awareness of abortion laws and services^7^, ^29^, ^32^, ^34^
Lack of knowledge about contraception and social norms regarding sons^22^, ^27^
Women resorting to covert and unsafe traditional procedures or self‐induced abortion^22^, ^37^
Abortions being performed for the wrong reasons^26^
Misuse of services, particularly by women not using family planning methods^26^
Sex‐selective abortions and abortions used as a family planning method^26^
Limited access to trained providers and equipment, particularly in remote areas^34^, ^35^, ^37^
Women's lack of knowledge about their legal rights^30^
Lack of counselors^28^
Lack of formal referral networks for women who have been denied abortion services elsewhere^29^
Lack of awareness of medical abortifacients among healthcare providers, prescription of varieties of allopathic and indigenous medicines by private providers and chemists for inducing abortion^36^

Facilitators improving access were categorized as: economic/geographic/societal; legal/policy; sociocultural; and healthcare systems factors (Table 2). The economic and geographical factors were the primary drivers in promoting accessibility to abortion services. The factors that were found to aid in the success of abortion services were the lowered cost, greater availability of SAS in rural communities, and the crucial roles played by auxiliary nurse‐midwives (ANM) and community health volunteers (CHV) in rural areas. The legal and policy facilitators included the legalization of abortion, government efforts to increase access to SAS, partnerships between government and NGOs, and increased awareness and education about abortion laws and services. Social and cultural facilitators included increased awareness and education about SAS, parental involvement in communication about sexual and reproductive health (SRH) with adolescents, availability of adolescent‐friendly healthcare services, confidential and nonjudgmental care provided by trained staff, supportive partners, friends, or family members, and the availability of information about SAS. The facilitators of the healthcare system included providing information and counseling to women seeking abortion care, training women CHV, increasing availability of medical abortion pills, pharmacy provision of abortion medications, training and support for non‐clinicians, expansion of SAS, training of providers, and established referral networks for women who have been denied abortion services elsewhere.

**Table 2:** Facilitators and their categories.

Facilitators
Economic, geographic, and societal factors
Reduction in the cost of abortion services^24^
Improving access to SAS in rural areas^7^
Improved access to SAS in rural areas^7^, ^24^
Participation of auxiliary nurse midwives, and community health volunteers in rural areas^33^
Legal and policy factors
Legalization of abortion^25^, ^38^
Government efforts to increase access to SAS^33^
Partnerships between government and NGOs to increase awareness and access to SAS^33^
Improved awareness and education about abortion laws and services^26^
Social and cultural factors
Increased awareness and education about SAS^7^, ^24^, ^25^, ^33^
Parental involvement in communication about SRH with adolescents^23^
Availability of adolescent‐friendly health services^23^
Confidential and nonjudgmental care provided by trained staff^23^
Supportive partners, friends, or family members, and the availability of information^32^
Positive views of healthcare staff and abortion service providers toward the new abortion law and practice^26^
Healthcare system factors
Providing information and counseling to women seeking abortion care^24^
Training female community health volunteers to provide information and referrals^28^
Increased availability of medical abortion pills^25^, ^33^, ^38^
Pharmacy provision of medical abortion^9^, ^33^, ^35^
Training and support for nonphysician clinicians^33^, ^35^
Expansion of SAS and provider training^7^, ^25^
Established referral networks for women who have been denied abortion services elsewhere^29^
Consistent access to medication for abortion^29^
Improved training for providers^29^
Increased confidentiality and privacy in healthcare settings^7^, ^24^
Reduced stigma surrounding abortion^7^
Support from local and international organizations to improve access to SAS^25^
Training and mobilization of mid‐level health workers, such as auxiliary nurse midwives^31^
Human resources (trained ANM) and contraceptive supplies^22^
Ongoing monitoring and evaluation of SAS to ensure that they are effective and equitable^9^

Abbreviations: ANM, auxiliary nurse‐midwives; NGO, nongovernmental organization; SAS, safe abortion services; SRH, sexual and reproductive health.

We analyzed the patterns and trends of barriers and facilitators, grouping them under legal/policy, sociocultural, geographic/accessibility, and healthcare systems factors (Table 3).

**Table 3:** Summary table showing the patterns and trends of barriers and facilitators for safe abortion services in Nepal.

**Factors**	Barriers	Facilitators
Legal and policy factors	Legal restrictions and policy barriers	Increasing support and cooperation between government and NGOs
	Limited access to necessary documentation and approvals	Improvements in awareness and education about abortion laws and services
	Strict legal sanctions against abortion	Increasing implementation of the new abortion law and services
Social and cultural factors	Stigma and discrimination against women	Increasing involvement of family and community members in communication
	Sociocultural beliefs and gender‐based factors	Increasing the availability of adolescent‐friendly health services
	Lack of awareness and knowledge about safe abortion services	Reduction in stigma surrounding abortion and more positive views
Geographic and accessibility factors	Limited availability and accessibility of safe abortion services	Ensuring safe abortion services accessible in rural areas
	Distance and transportation barriers	Increasing awareness and provision of information and counseling
	Lack of access to trained providers and equipment	Cost reduction and improved training for providers
Healthcare system factors	Limited infrastructure and resources in rural areas	Expansion and improvements in safe abortion services and training
	Inadequate knowledge and experience about the services required	Increasing availability and access to medical abortion pills
	High cost of abortion services	Improvements in confidentiality, privacy, and support for clinicians

Abbreviation: NGO, nongovernmental organization.

## 4 DISCUSSION

Our comprehensive review of barriers and facilitators revealed four predominant factors impacting access to safe and legal abortion services in Nepal. These interrelated factors span across multiple spheres and require coordinated strategies.

### 4.1 Legal and policy factors

In various studies, a common barrier that was consistently found was a lack of understanding of abortion laws and service availability.^7^, ^8^, ^23^, ^25^, ^32^, ^34^, ^37^, ^38^ This leaves women unaware of their rights and options,^8^ which ultimately leads to uncertainty, fear of legal repercussions, and stigma. Additionally, the spread of misinformation exacerbates misconceptions.^7^, ^8^

Strict legal restrictions pose significant challenges, making it very difficult for women to freely make choices about their own bodies and reproductive health.^24^, ^34^, ^37^ Particularly, in lower‐income settings such as Nepal, restrictive legal status increases disparities in accessing safe abortion care.^35^ Thankfully, the policy landscape has changed over the past two decades as abortion laws have become more liberal.^25^, ^38^ This has led to a significant shift toward recognizing women's reproductive health and that abortion is their fundamental right.

Despite these advances in the legal framework, barriers to access to abortion care remain. The policy requiring women who live far from healthcare facilities to obtain physician approval for medical abortion is an example of barriers that disproportionately affect rural populations.^28^ This restriction may increase inequalities. However, its recent amendment allows community healthcare workers to provide medical abortion, a promising policy change to expand access.^17^

It is critical to comprehensively address the complex legal and policy barriers to protect and uphold women's reproductive health and rights. To achieve equitable and safe access to abortion, it is important that strategies not only address ongoing restrictions, but also tackle existing awareness gaps and inequities. This requires coordinated efforts across sectors to establish an enabling environment for all individuals to exercise their right to abortion.^33^

Key facilitators such as legalizing abortion were transformative in decriminalizing the procedure and acknowledging women's autonomy.^25^, ^38^ Government–NGO partnerships have successfully increased awareness and availability of services.^33^ Better education on abortion laws and services also empowers women by giving them accurate information about their rights and options.^26^

### 4.2 Social and cultural factors

In Nepal, deeply rooted societal customs, values, and mindsets continue to uphold a harmful stigma surrounding abortion, impeding women's ability to access safe and necessary services.^8^, ^23^, ^24^, ^25^, ^28^, ^30^, ^32^, ^33^, ^34^, ^38^ This stigma originates from deeply ingrained patriarchal beliefs of women's morals and sexuality, which makes abortion taboo in our culture.^34^ It manifests itself through social isolation, verbal abuse, denial of services, and community disapproval of women seeking abortion.^23^, ^24^ Beyond emotional distress, stigma deters timely care‐seeking, compels unsafe procedures, and hinders women's ability to reach/afford services.^32^, ^33^, ^38^

Persistent gender inequities and limited control over reproductive decisions act as catalysts for perpetuation of stigma.^34^ As a result, women are left with few options and often resort to unsafe and secretive methods of abortion.^34^ Adding to this cycle, healthcare providers perpetuate stigma through their discriminatory actions and refusal to offer essential services.^8^, ^30^ Women encounter multilayered stigma originating from communities and the healthcare system.^23^, ^24^ Addressing stigma requires comprehensive strategies that span sociocultural, legal, health systems, and geographic spheres. Increased awareness, community engagement, improved provider training, and more robust legal frameworks can slowly transform social attitudes and norms.^7^, ^24^, ^25^, ^33^ Including parents in communicating about SRH with adolescents fosters supportive family environments.^23^ Adolescent‐friendly healthcare services meet the needs of young women by providing a welcoming space for information and care.^23^ Confidential, nonjudgmental care from trained staff mitigates stigma and makes women more likely to seek timely, appropriate care.^32^ Positive attitudes among healthcare staff toward abortion laws create an atmosphere of empathy and understanding that encourages care‐seeking.^26^

### 4.3 Geographic and accessibility factors

Nepal's diverse terrain creates geographic barriers that, along with economic and social factors, challenge access to SAS and underscore disparities.^8^, ^9^, ^25^, ^32^ Rural areas suffer from scarce facilities and trained providers, obstructing services.^8^, ^9^, ^25^, ^32^ Rugged topography poses transportation obstacles, with remote regions especially affected.^30^, ^33^ Many women struggle to afford travel and clinic fees, creating financial barriers.^8^, ^34^ Rural populations have limited access to SAS availability in general.^7^, ^24^ High private clinic costs further constrain the affordability.^7^, ^23^, ^24^, ^25^, ^30^, ^33^ These intertwined geographic, economic, and social barriers hinder timely and appropriate care.

However, some facilitators show promise in improving access. Reducing service costs alleviates financial limitations.^24^ Expanding availability beyond cities helps address geographic disparities in rural areas.^7^, ^24^ Leveraging local auxiliary nurses, midwives, and volunteers facilitates community‐level care.^33^

### 4.4 Healthcare system factors

Numerous barriers within Nepal's healthcare system obstruct the provision of SAS, requiring careful attention to pave the way for improved accessibility and quality.^7^, ^8^, ^22^, ^25^, ^30^, ^33^, ^34^, ^35^, ^38^ A major challenge is the lack of knowledge about abortion and available services among women.^8^, ^33^ Being uninformed hinders their ability to make appropriate decisions, potentially delaying care or leading to unsafe practices. The shortage of trained healthcare providers equipped to offer SAS results in inadequate counseling and care.^34^ The absence of a safe and confidential environment can deter women from seeking SAS, perpetuating unsafe abortions.^7^, ^23^, ^37^ The scarcity of trained providers and facilities, especially in certain regions, forces women to endure logistical hurdles to access care far from home.^8^, ^30^, ^38^ This underscores the urgency of expanding the reach of services to remote areas. Failure to provide effective postabortion contraception contributes to repeat unintended pregnancies and unsafe practices.^34^ The provision of inaccurate information and unsafe medications provided by untrained pharmacy staff further endangers women's health and lacks a proper referral system.^26^, ^35^

Despite these challenges, numerous facilitators show promise in improving SAS in Nepal. Providing women with comprehensive counseling and information promotes informed choices and timely, safe care.^24^ Training female CHV to offer referrals bridges the access gap, particularly in rural areas.^28^ Widening availability of medical abortion pills enables convenient, safe termination of early pregnancies.^25^, ^33^, ^38^ Pharmacies can serve as accessible points to obtain these medications privately.^9^, ^33^, ^35^ Training non‐clinicians in abortion care enhance access in underserved regions.^29^, ^33^, ^35^ Expanding the number of trained providers and facilities ensures that the services are within reach for more women.^7^, ^25^ Referral networks prevent denial of care and resorting to unsafe means.^29^ Fostering confidential, nonjudgmental settings encourages care‐seeking.^7^, ^24^ Reducing stigma creates a supportive environment.^7^ Collaborations with organizations provide resources to strengthen SAS delivery.^25^ Training mid‐level health providers such as ANM brings care closer to the communities.^31^ Ongoing monitoring and evaluation maintain quality and equity.^9^

### 4.5 Strengths and limitations

This review has both strengths and limitations. A major strength is the comprehensive search across multiple databases and gray literature, providing a solid foundation of evidence. The included studies utilized sound methodologies, including surveys, interviews, and record reviews. However, the quality and depth of evidence varied. Some barriers/facilitators were only supported by a few studies, indicating a need for more research in those areas. Many studies relied on self‐reported data, which could introduce bias. Most of the evidence came from cross‐sectional studies, limiting the ability to infer causality between barriers/facilitators and access outcomes. There was minimal data directly linking identified factors to abortion morbidity/mortality. Few studies evaluated interventions or policies to improve access. While valuable insights emerged, higher‐quality longitudinal and interventional studies are required to strengthen the knowledge base on improving the safe provision of abortion in Nepal.

## 5 CONCLUSION

The review identified and analyzed various barriers and facilitators to ensuring wider accessibility to SAS. The major barriers identified were financial constraints, unfavorable geography, lack of infrastructure, and fear of social stigmatization. The main factors favorable for abortion services were economics and geography. Legalization of abortion reinforced with improved access to abortion services in rural areas, reduced cost and the active involvement of ANM and CHV have played crucial roles in facilitating abortion services.

## AUTHOR CONTRIBUTIONS

**Alok Atreya**: Conceptualization; data curation; formal analysis; methodology; validation; visualization; writing—original draft; writing—review and editing. **Kishor Adhikari**: Writing—original draft; writing—review and editing. **Samata Nepal**: Data curation; formal analysis; investigation; methodology; writing—review and editing. **Milan Bhusal**: Writing—original draft. **Ritesh G. Menezes**: Methodology; resources; supervision; writing—review and editing. **Dhan B. Shrestha**: Project administration, resources, writing—review and editing. **Deepak Shrestha**: Writing – review and editing.

## CONFLICT OF INTEREST STATEMENT

The authors declare no conflict of interest.

## TRANSPARENCY STATEMENT

The lead author Alok Atreya affirms that this manuscript is an honest, accurate, and transparent account of the study being reported; that no important aspects of the study have been omitted; and that any discrepancies from the study as planned (and, if relevant, registered) have been explained.

## Supporting information

Supporting information.

## Data Availability

Our study did not generate any unique datasets or data files. All data used in this research are either publicly available in the manuscript or can be obtained through the referenced sources.

## References

[1] National Academies of Sciences, Engineering, and Medicine; Health and Medicine Division; Board on Health Care Services; Board on Population Health and Public Health Practice; Committee on Reproductive Health Services: Assessing the Safety and Quality of Abortion Care in the U.S . The Safety and Quality of Abortion Care in the United States. National Academies Press; 2018. 10.17226/24950 29897702

[2] Calvert C , Owolabi OO , Yeung F , et al. The magnitude and severity of abortion‐related morbidity in settings with limited access to abortion services: a systematic review and meta‐regression. BMJ Glob Health. 2018;3:e000692. 10.1136/bmjgh-2017-000692 PMC6035513 29989078

[3] Haddad LB , Nour NM . Unsafe abortion: unnecessary maternal mortality. Rev Obstet Gynecol. 2009;2:122‐126. 19609407 PMC2709326

[4] Yogi A , K.C P , Neupane S . Prevalence and factors associated with abortion and unsafe abortion in Nepal: a nationwide cross‐sectional study. BMC Pregnancy Childbirth. 2018;18:376. 10.1186/s12884-018-2011-y 30223798 PMC6142400

[5] Wu W‐J , Maru S , Regmi K , Basnett I . Abortion care in Nepal, 15 years after legalization: gaps in access, equity, and quality. Health Hum Rights. 2017;19:221‐230. 28630554 PMC5473051

[6] Mehata S , Menzel J , Bhattarai N , et al. Retracted article: factors associated with induced abortion in Nepal: data from a nationally representative population‐based cross‐sectional survey. Reprod Health. 2019;16:68. 10.1186/s12978-019-0732-7 31138253 PMC6540427

[7] Henderson JT , Puri M , Blum M , et al. Effects of abortion legalization in Nepal, 2001‐2010. PLoS One. 2013;8(5):e64775. 10.1371/journal.pone.0064775 23741391 PMC3669364

[8] Puri M , Singh S , Sundaram A , Hussain R , Tamang A , Crowell M . Abortion incidence and unintended pregnancy in Nepal. Int Perspect Sex Reprod Health. 2016;42:197‐209. 10.1363/42e2116 28825899 PMC5568822

[9] Rogers C , Sapkota S , Tako A , Dantas JAR . Abortion in Nepal: perspectives of a cross‐section of sexual and reproductive health and rights professionals. BMC Womens Health. 2019;19:40. 10.1186/s12905-019-0734-1 30808340 PMC6390627

[10] Tamang A . Women in Prison in Nepal for Abortion: A Study on Implications of Restrictive Abortion Law on Women's Social Status and Health. Center for Research on Environment, Health and Population Activities (CREHPA); 2000.

[11] World Health Organization . Unsafe Abortion Nepal country Profile. Ministry of Health and Population. 2006.

[12] National safe abortion policy . Ministry of Health and Population. 2003.

[13] Samandari G , Wolf M , Basnett I , Hyman A , Andersen K . Implementation of legal abortion in Nepal: a model for rapid scale‐up of high‐quality care. Reprod Health. 2012;7:9. 10.1186/1742-4755-9-7 PMC3373381 22475782

[14] Dhakal K , Ghimire S , Adhikari R . Effect of mass media on awareness of abortion law among women in Nepal. Tribhuvan Univ J. 2022;37:30‐41. 10.3126/tuj.v37i1.48210

[15] Atreya A , Shrestha M , Acharya J , Gurung S . Nepal—exploited by older married man—young unmarried mother accused of infanticide. Med Leg J. 2019;87:127‐129. 10.1177/0025817218822009 31179834

[16] The Right to Safe Motherhood and Reproductive Health Act , 2075 (2018). 2018.

[17] Annual Report 2077/78 (2020/21). 2022.

[18] Pant PD , Suvedi BK , Pradhan A , Hulton L , Matthew Z , Maskey M . Improvements in maternal health in Nepal: Further analysis of the 2006 Nepal Demographic and Health Survey. Macro International; 2008.

[19] Ministry of Health and Population, National Statistics Office . National population and housing census 2021: Nepal maternal mortality study 2021. 2022.

[20] Maistrellis E , Juma K , Khanal A , et al. Beyond abortion: impacts of the expanded global gag rule in Kenya, Madagascar and Nepal. BMJ Glob Health. 2022;7:7e008752. 10.1136/bmjgh-2022-008752 PMC9301792 35853673

[21] Ravaoarisoa L , Razafimahatratra MJJ , Rakotondratsara MA , et al. Slowing progress: the US global gag rule undermines access to contraception in Madagascar. Sex Reprod Health Matters. 2020;28:1838053. 10.1080/26410397.2020.1838053 33054631 PMC7887949

[22] KC NP , Basnett I , Sharma SK , Bhusal CL , Parajuli RR , Anderson KL . Increasing access to safe abortion services through auxiliary nurse midwives trained as skilled birth attendants. Kathmandu Univ Med J (KUMJ). 2012;9:260‐266. 10.3126/kumj.v9i4.6341 22710535

[23] Bhatta BR , Kiriya J , Shibanuma A , Jimba M . Parent–adolescent communication on sexual and reproductive health and the utilization of adolescent‐friendly health services in Kailali, Nepal. PLoS One. 2021;16:e0246917. 10.1371/journal.pone.0246917 33606727 PMC7894935

[24] Khatri RB , Poudel S , Ghimire PR . Factors associated with unsafe abortion practices in Nepal: pooled analysis of the 2011 and 2016 Nepal demographic and health surveys. PLoS One. 2019;14:e0223385. 10.1371/journal.pone.0223385 31596879 PMC6785064

[25] Lamichhane P , Harken T , Puri M , et al. Sex‐selective abortion in Nepal: a qualitative study of health workers' perspectives. Women's Health Issues. 2011;21:S37‐S41. 10.1016/j.whi.2011.02.001 21530837

[26] Möller A , Öfverstedt S , Siwe K . Proud, not yet satisfied: the experiences of abortion service providers in the Kathmandu valley, Nepal. Sex Reprod Healthc. 2012;3:135‐140. 10.1016/j.srhc.2012.10.003 23182445

[27] Ojha N , Sharma S , Paudel J . Post legalisation challenge: minimizing complications of abortion. Kathmandu Univ Med J (KUMJ). 2004;2:131‐136. 15821380

[28] Puri M , Tamang A , Shrestha P , Joshi D . The role of auxiliary nurse‐midwives and community health volunteers in expanding access to medical abortion in rural Nepal. Reprod Health Matters. 2014;22:94‐103. 10.1016/S0968-8080(14)43784-4 25702073

[29] Puri MC , Raifman S , Khanal B , Maharjan DC , Foster DG . Providers' perspectives on denial of abortion care in Nepal: a cross sectional study. Reprod Health. 2018;15(1):170. 10.1186/s12978-018-0619-z 30305079 PMC6180519

[30] Puri M , Vohra D , Gerdts C , Foster DG . “I need to terminate this pregnancy even if it will take my life”: a qualitative study of the effect of being denied legal abortion on women's lives in Nepal. BMC Womens Health. 2015;15:85. 10.1186/s12905-015-0241-y 26466784 PMC4606998

[31] Puri M , Regmi S , Tamang A , Shrestha P . Road map to scaling‐up: translating operations research study's results into actions for expanding medical abortion services in rural health facilities in Nepal. Health Res Policy Syst. 2014;12:24. 10.1186/1478-4505-12-24 24886393 PMC4030462

[32] Rocca C , Puri M , Dulal B , et al. Unsafe abortion after legalisation in Nepal: a cross‐sectional study of women presenting to hospitals. BJOG. 2013;120:1075‐1084. 10.1111/1471-0528.12242 23574112

[33] Rocca CH , Puri M , Shrestha P , et al. Effectiveness and safety of early medication abortion provided in pharmacies by auxiliary nurse‐midwives: a non‐inferiority study in Nepal. PLoS One. 2018;13:e0191174. 10.1371/journal.pone.0191174 29351313 PMC5774715

[34] Rogers C , Sapkota S , Paudel R , Dantas JAR . Medical abortion in Nepal: a qualitative study on women's experiences at safe abortion services and pharmacies. Reprod Health. 2019;16:105. 10.1186/s12978-019-0755-0 31307474 PMC6632190

[35] Samari G , Puri M , Cohen R , Blum M , Rocca CH . Pharmacy provision of medication abortion in Nepal: pharmacy owner and worker perspectives. Int Perspect Sex Reprod Health. 2018;44:81‐89. 10.1363/44e6518 30698524

[36] Tamang A , Tamang J . Availability and acceptability of medical abortion in Nepal: health care providers' perspectives. Reprod Health Matters. 2005;13:110‐119. 10.1016/S0968-8080(05)26194-3 16291492

[37] Thapa PJ , Thapa S , Shrestha N . A Hospital‐Based study of abortion in Nepal. Stud Fam Plann. 1992;23:311. 10.2307/1966528 1475798

[38] Singh S , Sundaram A , Hossain A , et al. Abortion service provision in south Asia: a comparative study of four countries. Contraception. 2020;102:210‐219. 10.1016/j.contraception.2020.05.015 32479764

